# Khat Chewing Habits in the Population of the Jazan Region, Saudi Arabia: Prevalence and Associated Factors

**DOI:** 10.1371/journal.pone.0134545

**Published:** 2015-08-06

**Authors:** Mohamed Salih Mahfouz, Bahaa-eldin E. A. Rahim, Yahya M. H. Solan, Anwar M. Makeen, Rashad Mohammed Alsanosy

**Affiliations:** 1 Family and Community Medicine Department, Faculty of Medicine, Jazan University, Jazan, Saudi Arabia; 2 Population Health Research Unit, Medical Research Center, Jazan University, Jazan, Saudi Arabia; 3 Department of Primary Healthcare, Ministry of Health, Jazan, Saudi Arabia; 4 Substance Abuse Research Center, Jazan University, Jazan, Saudi Arabia; 5 University of Gezira, Gezira State, Wad-Medani, Sudan; Catholic University of Sacred Heart of Rome, ITALY

## Abstract

The use of khat (*Catha edulis*) is a major public health and social problem that is believed to be growing globally. The khat chewing habit is prevalent in all areas of the Jazan region, Kingdom of Saudi Arabia (KSA). However, few studies have been conducted at the community level to investigate the khat chewing habits in this area. This study was conducted with the aim of assessing the prevalence and predictors of khat chewing among the Jazan community population. A cross-sectional study was conducted with a sample (*n* = 4,500) of the Jizani population who attended primary heath care centers in Jazan region. The participants were selected using a two-stage cluster random sampling. A structured questionnaire was used for data collection. The overall lifetime prevalence of khat chewing was 33.2% (95% CI 31.8–34.7) and was significantly higher for males 42.2% (95% CI 40.4–43.9) than for females 11.3% (95% CI 9.6–13.1) (*P* < 0.001). Current khat chewers accounted for 28.7% (95% CI 27.4–30.1) of the population sampled; 36.9% (95% CI 35.2–38.6) of whom were males, which is a significantly higher percentage than the 8.7% (95% CI 7.3–10.4) of current khat chewers who were females (*P* < 0.001). The multivariate logistic regression analysis suggests that the most important independent predictors of khat chewing were having a friend who chewed khat (OR = 20.1, *P* < 0.001), participant's smoking status (OR) = 3.9, *P* < 0.001), friend's smoking status (OR = 2.2, *P* < 0.001), gender (OR = 2.2, *P* < 0.001) and educational level (OR = 1.5, *P* < 0.05). A large proportion of the Jizani populations chew khat. Government and non-governmental organizations NGOs should design and strengthen community prevention programs to curb the high prevalence of khat use.

## Background

Khat *(Catha edulis*) is a green shrub that grows along the khat belt, which extends from eastern to southern Africa and the south west of the Arabian peninsula[1]. The plant originated in Ethiopia and grows in Somalia, Kenya, Malawi, Uganda, Zimbabwe, Afghanistan, Tanzania, the Congo, Zambia, Yemen and Madagascar[2,3].

Most of the effect of chewing khat is thought to come from two phenylalkylamines, cathinone and cathine, which are structurally related to amphetamine. Similar to amphetamine, cathinone may release serotonin in the central nervous system. Both cathinone and amphetamine increase the activity of the dopaminergic and noradrenergic pathways[4].

Khat users frequently report increased levels of energy, alertness and self-esteem, sensations of elation, enhanced imaginative ability and a greater capacity for associating ideas. An improvement in the users’ ability to communicate has also been reported. Students have also indicated that they had a better ability to review lessons and improved performance in exams following khat use [5–7].

Although there are conflicting opinions as to whether khat, similar to amphetamines, can actually cause dependence [1], the literature suggests that it is associated with public health and social problems [8–15]. In addition to health problems, khat use is associated with time wasting because the amount of time spent by users on khat chewing is considerable. Additionally, another group of studies has demonstrated an association between heavy khat consumption and psychosis [16–21]

Despite the negative aspects of the khat chewing habit and legal restrictions, the khat chewing practice is considerably prevalent among significant proportions of Jazan population. The habit has a deep-rooted cultural role within Jazan communities, this evident from khat chewing sessions which are regarded as a tool for enhancing social interaction in the region. Jazan location adjacent to Yemen, where khat is cultivated and universally consumed, contributed significantly to the availability of khat in the region via border smuggling. Since 1957, when laws punishing khat chewing were first enacted, the Saudi government has produced extraordinary efforts to fight the khat chewing habit in the region. However, neither development programs in the Faifa Mountains, where khat is cultivated locally, nor draconic punishment have reduced the khat chewing rates in the region. [22].

A number of studies have demonstrated patterns of khat use among school and university students [23–26]. Studies assessing khat use among other sectors of the population are scant. The main objective of this study was to reach a reasonable estimate of the prevalence of khat chewing in the Jazan region and to investigate the different factors associated with this widespread habit.

## Material and Methods

### Study area

Jazan is one of the 13 administrative regions of Saudi Arabia, located on the farthest southwest of Saudi Arabia and is demarcated by longitude 42°43 E and latitude 16°17 N. It borders the Aseer region from the north and east, the Red Sea from the west 330 kilometers along the seacoast, and the Republic of Yemen from the south and southeast. The total area of the region is estimated to be 40,457 km^2^ and includes approximately 80 islands on the Red Sea, the most famous of which is Farasan, which covers approximately 752 km^2^. According to the 2010 Saudi Arabia national population census, the region is populated by approximately 1.5 million people. Jazan population is almost composed of a group of Arabian tribes, making the population fairly homogeneous in ethnicity, religion, and language.

### Study design and participants

This was an observational cross-sectional survey targeting the Jizani population of both genders, aged older than 12 years, who attended selected primary health care centers in May 2012.

### Sampling procedures

A sample of 4500 persons was calculated, which was based on an estimated prevalence of khat use in Jazan region of 48.7%[25], 95% confidence interval (C.I.), marginal error 2%, non-response rate 20%, and design effect of 1.5. Two-stage stratified random sampling was utilized. In the first stage, eight sub-regions were randomly selected from the 13 sub-regions comprising the Jazan region. The selected sub-regions were Jazan, Abu Areesh, Fifa, Al-Ardhah, Aldarb, Sabia, Samtah, and Frsan. In the second stage, four primary health care centers (PHCs) were randomly selected from each sub-region. To execute the sampling plan, sampling frames were prepared in consultation with health administration affairs and respective PHCs. Probability proportional to size sampling (PPS) was used to determine the number of participants in the different PHCs.

### Data collection

A standardized questionnaire was used for data collection. A pilot study was conducted on 50 subjects to fine-tune the questions and assess the study instrument's reliability before initiating the actual data collection. Internal consistency reliability using Cronbach’s coefficient alpha was assessed and produced a values of 0.74. After the pilot study was conducted, some modifications were made to the original questionnaire. The final questionnaire contained approximately 40 multiple-choice questions organized into two sections. The questions covered background socioeconomic and demographic data and khat chewing practice variables. The questionnaire asked the participant whether he/she had tried chewing khat at least once in their lifetime, whether they had tried it in the last month, daily khat use and intermittent use. These questions were used to evaluate the lifetime prevalence of khat use, and the current prevalence of khat chewing. Questions on friend’s khat chewing and smoking status were also included in the questionnaire and were based on life time smoking or khat chewing. The personal interview was conducted by health workers from the health directorate, who attended a special training workshop on the data collection methodology.

We used the following operational definitions: (a) non-khat user: participants who had never used khat in any form; (b) current prevalence of chewing: the proportion of the study population who had chewed khat in the 30 days preceding the study; (c) ever chewer: an individual who had chewed, even if only once, in his/her lifetime; and (d) intermittent khat chewer: a person who chewed khat from time to time, for example, limited khat chewing specific to certain contexts, such as social occasions, festivals, or often. Khat chewing pattern of use was also assessed during the 30 days preceding the survey and classified as follows; chewing khat once a time per day; two to three times per day and chewing khat more than three times per day.

### Data management and analysis

The data entry took place at Substance Abuse Research Center, Jazan University. The data entry and analysis were performed using Epi-info (version 3.5.3) and SPSS (version 17 Inc., Chicago, IL, USA) software. Descriptive statistics, as well as inferential statistics, were used for data analysis. Simple tabulation frequencies were used to give a general overview of the data. The prevalence of khat chewing was presented using 95% CIs, and the Chi-squared test was performed to determine the associations between individual categorical variables and outcome (khat chewing). Significant variables from the Chi square test were included in multivariate analysis using Binary Logistic Regression. The multivariate logistic regression model was derived through simultaneous entry analysis. Adjusted and unadjusted odds ratios (OR) and their 95% confidence intervals (CIs) were used as indicators of strength of association. The final logistic regression model was checked for fitness using the Hosmer-Lemeshow goodness of fit test. The model was also analyzed for all possible two-way interactions and revealed no significant interactions in the final model. A *P*-value of 0.05 or less was used as the cut-off level for statistical significance.

### Ethical considerations

This study was conducted in accordance with the ethical standards within the political borders of the Kingdom of Saudi Arabia. All participants, including the guardians on behalf of minors/children participants, who were involved in this study read, understood and signed a written consent form. This study was approved by the IRB committee of the Medical Research Center of Jazan University, Kingdom of Saudi Arabia. The participants were told that they had the freedom to participate or withdraw from the study at any time. The anonymity of participants was emphasized, and confidentiality was strictly maintained for all collected questionnaires.

## Results

The response rate for the survey was 95.67% (4,305 from the target of 4,500 participants). The response rates for answering particular questionnaire questions varied because some subjects did not answer some questions. As observed in Table 1, the majority of sampled participants (51.0%) belonged to the age group of 25–39 years. The distribution of the Jizani people according to mode of living showed that 42.4% of them were from rural areas, whereas 57.6% were from urban areas. The marital status distribution showed that 60.8% of them were married and 32.6% were single. The working status distribution showed that 48.1% were engaged in work, and 51.9% were not working.

**Table 1:** Some selected characteristics of the study participants.

Characteristics	Male	Female	Total
	No(%)	No(%)	No(%)
**Age Groups (*n* = 4224)**			
10–14 Years	33(1.1)	22(1.8)	55(1.3)
15–24 Years	684(22.8)	220(18.0)	904(21.4)
25–39 Years	1528(50.9)	625(51.1)	2153(51.0)
40–49 Years	434(14.5)	232(19.0)	666(15.8)
50–64 Years	262(8.7)	97(7.9)	359(8.5)
65+ Years	60(2.0)	27(2.2)	87(2.1)
**Educational Level (*n* = 4570)**			
Illiterate	182(6.0)	245(19.7)	427(9.9)
Primary	440(14.7)	244(19.6)	963(16.2)
Intermediate	589(19.3)	175(14.0)	764(17.8)
Secondary	1059(34.7)	266(21.3)	1325(30.8)
University and above	776(25.4)	361(25.3)	1091(16.2)
**Mode of Living (*n* = 4257)**			
Rural	1378(45.7)	426(34.3)	1804(42.4)
Urban	1638(54.3)	815(65.7)	2453(57.6)
**Marital Status (*n* = 4285)**			
Single	1047(34.4)	350(28.2)	1397(32.6)
Married	1927(63.3)	680(54.8)	2607(60.8)
Divorced	37(1.2)	127(10.2)	164(3.8)
Widowed	34(1.1)	83(6.7)	117(2.7)
**Working Status (*n* = 4175)**			
Working	1719(58.3)	289(23.6)	2008(48.1)
Not Working	1231(41.7)	936(76.4)	2167(51.9)
**Total**	3058(100)	(1247)(100)	4305(100)

As shown in Table 2, the prevalence of ever khat chewing among Jizani people was 33.2% (95% CI 31.8–34.7) and was significantly higher for males 42.2% (95% CI 40.4–43.9) than for females 11.3% (95% CI 9.6–13.1) (***P*** < 0.001). Current khat chewers accounted for 28.7% (95% CI 27.4–30.1) of the population sampled; of the total number of males, 36.9% (95% CI 35.2–38.6) were current khat chewers, which is a significantly higher percentage than the 8.7% (95% CI 7.3–10.4) of females who were current khat chewers (***P*** < 0.001). The intermittent khat chewing prevalence was found to be 8.5% among males and only 2.6% among females.

**Table 2:** Prevalence of khat chewing among the Jazan Population, according to gender.

	Gender				Total		
Khat chewing status	Male		Female				*P*. Value
	**No(%)**	**95% C.I.**	**No(%)**	**95% C.I.**	**No(%)**	**95% C.I.**	
Ever khat chewers	1290(42.2)	40.4–43.9	141(11.3)	9.6–13.1	1431(33.2)	31.8–34.7	**0.000**
Current khat chewers	1127(36.9)	35.2–38.6	109(8.7)	7.3–10.4	1236(28.7)	27.4–30.1	**0.000**
Intermittent khat chewers	257(8.5)	7.5–9.4	33(2.6)	1.9–3.8	290(6.7)	6.0–7.5	**0.000**

Table 3 shows the patterns of khat chewing among both male and female participants. It is clear from the table that the majority of the study sample, 69.3%, started chewing khat during the age group 15–24 years, while only 12.4% of them started chewing khat when they were less than 15 years of age, with a significant difference between male and female responses (***P***<0.001). Regarding the pattern of khat use, 33.0% of khat chewers reported the use of khat once a time per days. Approximately 30% used khat 1–3 times a week, whereas only 6.3% of them chewed khat two to three times a day, with significant differences between males and females (***P*** <0.001). Although khat is banned by the government, most users, 68.7%, argued that it is very easy or easy to obtain khat, though 30.7% said that it was difficult to obtain khat, with no significant difference observed between males and females (***P*** >0.05). Almost 79.7% of khat users bought khat directly from sellers. In total, 14.7% of males shared khat with their friends, compared with 24.8% of females (***P*** <0.001). The majority of khat users (90.1%) said that their motives for chewing khat was for fun, whereas only 5.3% chewed khat to follow their parents (***P*** <0.001).

**Table 3:** Pattern of khat chewing, according to gender.

Characteristics	Male	Female	Total	*P*. Value
**Age when started khat chewing**				**0.000**
12–14 years	166(13.0)	9(6.7)	175(12.4)	
15–24 years	911(71.4)	66(49.3)	977(69.3)	
25 years and above	199(15.6)	59(44.0)	258(18.3)	
**Khat chewing pattern**				**0.000**
Once a time per day	411(31.9)	59(43.4)	470(33.0)	
Two to three times a day	75(5.8)	15(11.0)	90(6.3)	
More than three times per day	137(10.6)	11(8.1)	148(10.4)	
One to three times a week	408(31.7)	18(13.2)	426(29.9)	
From time to time	275(20.0)	33(24.3)	290(20.4)	
**Is it easy to obtain khat?**				**0.593**
Very easy	323(25.1)	27(20.0)	350(24.6)	
Easy	561(43.6)	65(48.1)	626(44.1)	
Difficult	394(30.6)	42(31.1)	436(30.7)	
Impossible	8(.6)	1(.7)	9(.6)	
**From where do you obtain khat**				**0.000**
I buy it	1039(81.2)	87(65.4)	1126(79.7)	
I share it with my friends	188(14.7)	33(24.8)	221(15.7)	
I share it with my parents	26(2.0)	4(3.0)	30(2.1)	
I share it with my father	22(1.7)	6(4.5)	28(2.0(	
I share it with my mother	29(.2)	1(.8)	3(.2)	
Other	2(.2)	2(1.5)	4(.3)	
**Why do you chew khat?**				**0.000**
Just for fun	1110(90.2)	116(89.2)	1226(90.1)	
I follow my parents	38(3.8)	22(19.1)	60(5.3)	
I follow my friends	319(31.2)	61(53.5)	380(33.4)	
To release tension	464(45.4)	65(57.0)	529(46.6)	

Socio-demographic variables, such as participant's age, educational level, marital status and working status, were all found to be highly associated with participants' khat use (***P*** < 0.001 for all). The prevalence of khat chewing increased with increasing age, while the prevalence of khat chewing differed according to variations in educational status. Having a friend who chewed khat or smoked, as well as the smoking status of the study participant, were also positively associated with a student's khat chewing status (***P*** < 0.001 for all). Although the prevalence of khat chewing in rural areas was 34.6%, compared with 31.9% in the urban population, no significant difference was observed (***P*** < 0.05) (Table 4).

**Table 4:** Khat chewing prevalence, according to some selected characteristics.

Variables	Khat chewing Prevalence	95% C.I.	*P*. Value
**Age groups (n = 4224)**			**0.000**
**12–15 years**	**10(18.2)**	**10.2–30.4**	
**15–24 Years**	**221(24.4)**	**21.8–27.4**	
**25–39 Years**	**784(36.4)**	**34.4–38.5**	
**40–49 Years**	**245(36.8)**	**33.2–40.5**	
**50–64 Years**	**132(36.8)**	**31.9–41.9**	
**65+ Years**	**25(28.7)**	**20.3–39.0**	
**Educational level (n = 4301)**			**0.000**
**Illiterate**	**114(26.7)**	**22.7–31.1**	
**Primary**	**270(39.0)**	**35.4–42.6**	
**Intermediate**	**278(36.4)**	**33.1–39.9**	
**Secondary**	**467(35.2)**	**32.7–37.9**	
**University and Above**	**302(27.7)**	**25.1–30.4**	
**Mode of living (n = 4257)**			**0.060**
**Rural**	**624(34.6)**	**32.4–36.8**	
**Urban**	**783(31.9)**	**30.1–33.8**	
**Marital status (n = 4285)**			**0.000**
**Single**	**384(27.5)**	**25.2–29.9**	
**Married**	**966(37.1)**	**35.2–38.9**	
**Divorced**	**42(25.6)**	**19.5–32.8**	
**Widowed**	**30(25.6)**	**18.6–34.3**	
**Working status (n = 4175)**			**0.000**
**Working**	**823(41.0)**	**38.9–43.2**	
**Not working**	**556(25.7)**	**23.9–27.5**	
**Cigarette smoking status (n = 4305)**			**0.000**
**Yes**	**609(59.2)**	**56.2–62.2**	
**No**	**822(25.1)**	**23.6–26.6**	
**Friend’s smoking status(n = 3866)**			**0.000**
**Yes**	**1125(42.0)**	**40.1–43.1**	
**No**	**72(6.1)**	**4.9–7.6**	
**Friend using khat (n = 3924)**			**0.000**
**Yes**	**1245(45.0)**	**43.0–46.8**	
**No**	**25(2.2)**	**1.4–3.2**	

The results of the univariate and multivariate logistic regression analyses for potential risk factors of khat chewing are shown in Table 5. Univariate analysis revealed that gender, school level, participant's age and smoking status, employment status, having a friend who chew khat or smoke were associated with a significant risk for khat chewing (***P*** < 0.001 for all). The multivariate logistic regression analysis suggested that the most important independent predictors of khat chewing among our sample were friends using khat (OR = 20.1, ***P*** < 0.001), participant's smoking status (OR) = 3.9, ***P*** < 0.001), friend's smoking (OR = 2.2, ***P*** < 0.001), participant's gender (OR = 2. 2, ***P*** < 0.001) and educational level (OR = 1.5, ***P*** < 0.05).

**Table 5:** Univariate and multivariate logistic regression analyses for khat chewing-related factors among study participants.

Category	Univariate			Multivariate#		
	OR	95% C.I.	*P*. Value	OR	95% C.I.	*P*. Value
**Gender**						
Female*	1			1		
Male	5.7	4.7–6.9	0.000	2.2	1.7–2.9	0.000
**Educational level**						
Literate*	1			1		
Illiterate	1.1	1.04–1.18	0.002	1.5	1.0–2.0	0.033
**Age groups**						
12- 25years*	1			1		
25–44 years	1.6	1.3–1.9	0.000	1.4	1.0–1.9	0.061
45+years	0.8	0.71–1.0	0.052	0.9	0.7–1.2	0.592
**Working status**						
Not working*	1			1		
Working	2.0	1.8–2.3	0.000	1.1	1.0–2.0	0.227
**Marital status**						
Single*	1			1		
Married	0.64	0.56–74	0.000	1.9	1.0–3.7	0.060
Divorced	1.10	0.76–1.6	0.610	1.4	0.8–2.7	0.275
Widowed	1.1	0.71–1.7	0.667	1.1	0.5–2.3	0.870
**Participant smoking**						
No*	1			1		
Yes	4.34	3.7–5.03	0.000	3.9	3.2–4.7	0.000
**Friend’s smoking**						
No*	1					
Yes	11.1	8.7–14.3	0.000	2.2	1.7–3.0	0.000
**Friend using khat**						
No*	1			1		
Yes	36.8	24.6–55.1	0.000	20.1	12.4–32.5	0.000

* Reference category.

# Adjusted for other variables in the table.

## Discussion

The aim of this study was to explore the magnitude of khat abuse among the Jazan community and to reach a reasonable prevalence estimate, in addition to investigating the most important independent factors influencing khat use in the region. An important result was that a significant proportion of the Jizani population who chewed khat, as overall ever users and current users, accounted for 33.2% and 28.7% of the study population, respectively. There was a significant difference between males and females for both indicators. Lifetime khat chewers and current chewers accounted for 42.2% and 36.9% of males and 11.3% and 8.7% of females, respectively. If we compare our estimates with previous studies conducted in the Jazan region, specifically, the only estimate at the community level, which was produced by (Milaat, *et al*., 2005) [25], they showed a current khat chewing prevalence among males of 48.7%, which is much higher than our estimate (36.9%) among males. Our new estimate may indicate a slight decrease in the prevalence of khat chewing in Jazan region. Increased awareness towards the harmful effects of khat in addition to the efforts exerted by the governmental and non-governmental organizations working on khat prevention programs may be responsible for this decline.

Our estimates are in agreement with the recent estimates produced by two surveys conducted by the newly established Substance Abuse Research Centre (SARC) in Jazan University. The first survey [27], which was conducted among University students, showed a current prevalence rate of 38.5% among males. The second survey[23], which was conducted among school students, also showed current prevalence rates among males of 33.1%, with 35.8% and 33.5% among fathers and brothers, respectively[24]. Surprisingly, our estimate was also close to the results of the khat survey conducted seven years ago, which showed that the current khat chewing rate among school and college students was 37.7% [25, 28 and 29]. This quick comparison indicates that khat chewing is still prevalent in the Jazan region, despite the efforts exerted by the government over the past ten years. In comparison to other countries, our prevalence estimate of khat chewing prevalence in Jazan was less than that observed among the general populations in Somalia[30] and Yemen[31 and 32].

Although khat cultivation and trade is officially prohibited in the Kingdom of Saudi Arabia, the majority of the participants, almost 68.7%, stated that it is very easy or easy to obtain khat. Moreover, 79.7% of khat chewers bought khat directly from sellers. This finding indicates the availability of khat as commodity in the Jazan region. Previous studies in the Jazan region indicated the same pattern[27]. Several factors contribute to the availability of khat in this region. First, the Jazan region shares very long borders with Yemen, the global producer of khat. Additionally, across the Red Sea, in Ethiopia, Somalia and Kenya, khat production and trading is legal [33], and smuggling is difficult to control. Second, the Faifa mountain area in Jazan remains a producer of khat, although the KSA government prohibits the expansion of khat cultivation in the mountains and has established the Faifa Development Authority to control khat cultivation and support farmers in replacing khat with alternative cash crops [25]. The effectiveness of such interventions need to be assessed because still khat is available in Jazan. Third, in 2006, a new, stricter legal act banning khat was enacted, but this regulation was replaced by a new, more relaxed one, which discerns between khat and other drugs. This relaxation seems to have encouraged users, and many Jizani people attribute the recent high khat abuse rates to this rule.

Our study revealed that the motives behind khat chewing was for fun among almost 90.1% of study participants, whereas 46.6% reported the use of khat for releasing tension. This interpretation is consistent with the findings of other studies [34]. Unlike previous studies conducted in the region [24, 25 and 27], our study showed no significant difference between khat chewing patterns in the urban (31.9%) and rural areas (34.6%). Khat consumption was significantly higher among workers than non-workers, which was consistent with previous findings (Patel *et al*., 2005) [35]. A reasonable justification for this finding is that khat is an expensive commodity in Jazan and users allocate a substantial part of family income for khat consumption. A similar pattern has been noticed in other communities abusing khat [36].

The most significant independent risk factors of khat chewing identified in this study were having a friend who used khat (OR = 20.1), participant's smoking status (OR = 3.9), having a friend who smoked (OR = 2.2), and participant's gender (OR = 2.2). Peer influence is a very important factor that has been associated with risky behavior because individuals need a sense of belonging to social networks. This agrees with the findings from similar studies [24 and 37]. The present findings showed that participants who used tobacco were associated with an increased risk of khat chewing. Consistent with our findings, several studies have documented an association between cigarette smoking and substance use among users[38 and 39]. In this study, the habit of chewing khat by males was associated with an increased odds of chewing khat among the study participants. This might be due to the more common tendency for males to abuse substances, compared with females [40], and to the greater cultural acceptance of male substance use in Jazan region [24].

The strength of this study is that it focuses on the community level, whereas most published studies have been conducted among specialized groups of society, such as students. Despite this strength, the study has some limitations that should be mentioned to facilitate the proper understanding of study outcomes. First, because the work is based on a cross-sectional survey design, the direction of relationships and causal relationships cannot be determined. Second, the population of this study included only patients attending primary care centers of the Jazan region. This might have implications in the representativeness of the data. PHCs attendees are not fully representative of the general population because they are a specialized group. However, we relied on PHCs for our research because they are the only culturally accepted place for conducting such studies among the population, other than household surveys. Another point is that PHCs are well distributed in the Jazan province, thus allowing for systematic coverage of all parts of the region. Finally the study sample was biased towards males; this is because previous literature suggested high prevalence of khat chewing among males compared to females. Based on what we have mentioned in this section the results of this study should be interpreted with caution.

## Conclusion

A large proportion of the Jizani population are khat chewers. The consumption of khat was significantly associated with having a friend who chews khat or smokes, the participant's smoking status, and the gender of the participant. Government and non-governmental organizations (NGOs) should design and strengthen community prevention programs to curb the high prevalence of khat use. Law enforcement alone is not enough to reduce the high prevalence of this deeply rooted practice among the Jazan population.
